# SciPy 1.0: fundamental algorithms for scientific computing in Python

**DOI:** 10.1038/s41592-019-0686-2

**Published:** 2020-02-03

**Authors:** Pauli Virtanen, Ralf Gommers, Travis E. Oliphant, Matt Haberland, Tyler Reddy, David Cournapeau, Evgeni Burovski, Pearu Peterson, Warren Weckesser, Jonathan Bright, Stéfan J. van der Walt, Matthew Brett, Joshua Wilson, K. Jarrod Millman, Nikolay Mayorov, Andrew R. J. Nelson, Eric Jones, Robert Kern, Eric Larson, C J Carey, İlhan Polat, Yu Feng, Eric W. Moore, Jake VanderPlas, Denis Laxalde, Josef Perktold, Robert Cimrman, Ian Henriksen, E. A. Quintero, Charles R. Harris, Anne M. Archibald, Antônio H. Ribeiro, Fabian Pedregosa, Paul van Mulbregt, SciPy 1.0 Contributors, Aditya Vijaykumar, Alessandro Pietro Bardelli, Alex Rothberg, Andreas Hilboll, Andreas Kloeckner, Anthony Scopatz, Antony Lee, Ariel Rokem, C. Nathan Woods, Chad Fulton, Charles Masson, Christian Häggström, Clark Fitzgerald, David A. Nicholson, David R. Hagen, Dmitrii V. Pasechnik, Emanuele Olivetti, Eric Martin, Eric Wieser, Fabrice Silva, Felix Lenders, Florian Wilhelm, G. Young, Gavin A. Price, Gert-Ludwig Ingold, Gregory E. Allen, Gregory R. Lee, Hervé Audren, Irvin Probst, Jörg P. Dietrich, Jacob Silterra, James T Webber, Janko Slavič, Joel Nothman, Johannes Buchner, Johannes Kulick, Johannes L. Schönberger, José Vinícius de Miranda Cardoso, Joscha Reimer, Joseph Harrington, Juan Luis Cano Rodríguez, Juan Nunez-Iglesias, Justin Kuczynski, Kevin Tritz, Martin Thoma, Matthew Newville, Matthias Kümmerer, Maximilian Bolingbroke, Michael Tartre, Mikhail Pak, Nathaniel J. Smith, Nikolai Nowaczyk, Nikolay Shebanov, Oleksandr Pavlyk, Per A. Brodtkorb, Perry Lee, Robert T. McGibbon, Roman Feldbauer, Sam Lewis, Sam Tygier, Scott Sievert, Sebastiano Vigna, Stefan Peterson, Surhud More, Tadeusz Pudlik, Takuya Oshima, Thomas J. Pingel, Thomas P. Robitaille, Thomas Spura, Thouis R. Jones, Tim Cera, Tim Leslie, Tiziano Zito, Tom Krauss, Utkarsh Upadhyay, Yaroslav O. Halchenko, Yoshiki Vázquez-Baeza

**Affiliations:** 1 University of Jyväskylä, Jyväskylä, Finland; 2 Quansight LLC, Austin, TX USA; 3 Ultrasound Imaging, Mayo Clinic, Rochester, MN USA; 4 Electrical Engineering, Brigham Young University, Provo, UT USA; 5 Enthought, Inc., Austin, TX USA; 6 Anaconda Inc., Austin, TX USA; 7 BioResource and Agricultural Engineering Department, California Polytechnic State University, San Luis Obispo, CA USA; 8 Department of Mathematics, University of California Los Angeles, Los Angeles, CA USA; 9 Los Alamos National Laboratory, Los Alamos, NM USA; 10 Independent researcher, Tokyo, Japan; 11 National Research University Higher School of Economics, Moscow, Russia; 12 Independent researcher, Saue, Estonia; 13 Department of Mechanics and Applied Mathematics, Institute of Cybernetics at Tallinn Technical University, Tallinn, Estonia; 14 Berkeley Institute for Data Science, University of California Berkeley, Berkeley, CA USA; 15 Independent researcher, New York, NY USA; 16 School of Psychology, University of Birmingham, Edgbaston, Birmingham UK; 17 Independent researcher, San Francisco, CA USA; 18 Division of Biostatistics, University of California Berkeley, Berkeley, CA USA; 19 WayRay LLC, Skolkovo Innovation Center, Moscow, Russia; 20 Australian Nuclear Science and Technology Organisation, Lucas Heights, NSW Australia; 21 Institute for Learning and Brain Sciences, University of Washington, Seattle, WA USA; 22 College of Information and Computing Sciences, University of Massachusetts Amherst, Amherst, MA USA; 23 Independent researcher, Amsterdam, the Netherlands; 24 Berkeley Center for Cosmological Physics, University of California Berkeley, Berkeley, CA USA; 25 Bruker Biospin Corp., Billerica, MA USA; 26 University of Washington, Seattle, WA USA; 27 Independent researcher, Toulouse, France; 28 Independent researcher, Montreal, Quebec Canada; 29 New Technologies Research Centre, University of West Bohemia, Plzeň, Czech Republic; 30 Department of Mathematics, Brigham Young University, Provo, UT USA; 31 Oden Institute for Computational Engineering and Sciences, The University of Texas at Austin, Austin, TX USA; 32 Independent researcher, Belmont, Massachusetts USA; 33 Space Dynamics Laboratory, North Logan, UT USA; 34 Independent researcher, Logan, Utah USA; 35 Anton Pannekoek Institute, Amsterdam, The Netherlands; 36 Graduate Program in Electrical Engineering, Universidade Federal de Minas Gerais, Belo Horizonte, Brazil; 37 Google LLC, Montreal, Quebec Canada; 38 Google LLC, Cambridge, MA USA; 40 International Centre for Theoretical Sciences, Tata Institute of Fundamental Research, Bengaluru, India; 41 Department of Physics, Birla Institute of Technology and Science, Pilani, India; 42 Independent researcher, Milan, Italy; 43 Institute of Environmental Physics, University of Bremen, Bremen, Germany; 44 Department of Computer Science, University of Illinois at Urbana-Champaign, Urbana, IL USA; 45 Laboratoire Photonique, Numérique et Nanosciences UMR 5298, Université de Bordeaux, Institut d’Optique Graduate School, CNRS, Talence, France; 46 The University of Washington eScience Institute, The University of Washington, Seattle, WA USA; 47 Federal Reserve Board of Governors, Washington, DC, USA; 48 Datadog Inc., New York, NY USA; 49 HQ, Orexplore, Stockholm, Sweden; 50 Statistics Department, University of California - Davis, Davis, CA USA; 51 Emory University, Atlanta, GA USA; 52 Applied BioMath, Concord, MA USA; 53 Department of Computer Science, University of Oxford, Oxford, UK; 54 NeuroInformatics Laboratory, Bruno Kessler Foundation, Trento, Italy; 55 Independent researcher, Chicago, IL USA; 56 Department of Engineering, University of Cambridge, Cambridge, UK; 57 Aix Marseille Univ, CNRS, Centrale Marseille, LMA, Marseille, France; 58 Interdisciplinary Center for Scientific Computing (IWR), Heidelberg University, Heidelberg, Germany; 59 ABB Corporate Research, ABB AG, Ladenburg, Germany; 60 Institut für Mathematische Optimierung, Technische Universität Carolo-Wilhelmina zu Braunschweig, Braunschweig, Germany; 61 Independent researcher, Cologne, Germany; 62 Lawrence Berkeley National Laboratory, Berkeley, CA USA; 63 Institut für Physik, Universität Augsburg, Augsburg, Germany; 64 Applied Research Laboratories, The University of Texas at Austin, Austin, TX USA; 65 Department of Radiology, School of Medicine, University of Cincinnati, Cincinnati, OH USA; 66 Department of Radiology, Cincinnati Children’s Hospital Medical Center, Cincinnati, OH USA; 67 Ascent Robotics Inc., Tokyo, Japan; 68 ENSTA Bretagne, Brest, France; 69 Faculty of Physics, Ludwig-Maximilians-Universität, München, Germany; 70 Excellence Cluster Universe, München, Germany; 71 Independent researcher, Malden, Massachusetts USA; 72 Data Sciences, Chan Zuckerberg Biohub, San Francisco, CA USA; 73 Faculty of Mechanical Engineering, University of Ljubljana, Ljubljana, Slovenia; 74 Sydney Informatics Hub, The University of Sydney, Camperdown, NSW Australia; 75 Instituto de Astrofísica, Pontificia Universidad Católica de Chile, Santiago, Chile; 76 Max Planck Institute for Extraterrestrial Physics, Garching, Germany; 77 University of Stuttgart, Machine Learning and Robotics Lab, Stuttgart, Germany; 78 Department of Electrical Engineering, Universidade Federal de Campina Grande, Campina Grande, Brazil; 79 Department of Computer Science, Kiel University, Kiel, Germany; 80 Planetary Sciences Group and Florida Space Institute and Department of Physics, University of Central Florida, Orlando, FL USA; 81 Independent researcher, Madrid, Spain; 82 Monash Micro Imaging, Monash University, Clayton, VIC Australia; 83 Department of Molecular, Cellular, and Developmental Biology, University of Colorado, Boulder, Boulder, CO USA; 84 Department of Physics and Astronomy, Johns Hopkins University, Baltimore, MD USA; 85 Independent researcher, Munich, Germany; 86 Center for Advanced Radiation Sources, The University of Chicago, Chicago, IL USA; 87 University of Tübingen, Tübingen, Germany; 88 Independent researcher, Rugby, UK; 89 Two Sigma Investments, New York, NY USA; 90 Department of Mechanical Engineering, Technical University of Munich, Garching, Germany; 91 Independent researcher, Berkeley, CA USA; 92 Independent researcher, London, UK; 93 Independent researcher, Berlin, Germany; 94 Intel Corp., Austin, TX USA; 95 Independent Researcher, Horten, Norway; 96 Independent researcher, Daly City, CA USA; 97 D. E. Shaw Research, New York, NY USA; 98 Division of Computational Systems Biology, Department of Microbiology and Ecosystem Science, University of Vienna, Vienna, Austria; 99 Independent researcher, Melbourne, Australia; 100 School of Physics and Astronomy, University of Manchester, Manchester, UK; 101 Electrical and Computer Engineering, University of Wisconsin–Madison, Madison, WI USA; 102 Dipartimento di Informatica, Università degli Studi di Milano, Milan, Italy; 103 Inter-University Centre for Astronomy and Astrophysics, Ganeshkhind, Pune, India; 104 Kavli Institute for the Physics and Mathematics of the Universe, Kashiwa-shi, Japan; 105 Waymo LLC, Mountain View, CA USA; 106 Faculty of Engineering, Niigata University, Nishi-ku, Niigata, Japan; 107 Virginia Polytechnic Institute and State University, Blacksburg, VA USA; 108 Aperio Software, Headingley Enterprise and Arts Centre, Leeds, UK; 109 Independent researcher, Duisburg, Germany; 110 Broad Institute, Cambridge, MA USA; 111 Independent researcher, Gainesville, FL USA; 112 Department of Psychology, Humboldt University of Berlin, Berlin, Germany; 113 Epiq Solutions, Schaumburg, IL USA; 114 Max Planck Institute for Software Systems, Kaiserslautern, Germany; 115 Department of Psychology and Brain Sciences, Dartmouth College, Hanover, NH USA; 116 Jacobs School of Engineering, University of California San Diego, La Jolla, CA USA

**Keywords:** Computational biology and bioinformatics, Biophysical chemistry, Technology

## Abstract

SciPy is an open-source scientific computing library for the Python programming language. Since its initial release in 2001, SciPy has become a de facto standard for leveraging scientific algorithms in Python, with over 600 unique code contributors, thousands of dependent packages, over 100,000 dependent repositories and millions of downloads per year. In this work, we provide an overview of the capabilities and development practices of SciPy 1.0 and highlight some recent technical developments.

## Abstract (web summary)

This Perspective describes the development and capabilities of SciPy 1.0, an open source scientific computing library for the Python programming language.

## Main

SciPy is a library of numerical routines for the Python programming language that provides fundamental building blocks for modeling and solving scientific problems. SciPy includes algorithms for optimization, integration, interpolation, eigenvalue problems, algebraic equations, differential equations and many other classes of problems; it also provides specialized data structures, such as sparse matrices and *k*-dimensional trees. SciPy is built on top of NumPy^1,2^, which provides array data structures and related fast numerical routines, and SciPy is itself the foundation upon which higher level scientific libraries, including scikit-learn^3^ and scikit-image^4^, are built. Scientists, engineers and others around the world rely on SciPy. For example, published scripts^5,6^ used in the analysis of gravitational waves^7,8^ import several subpackages of SciPy, and the M87 black hole imaging project cites SciPy^9^.

Recently, SciPy released version 1.0, a milestone that traditionally signals a library’s API (application programming interface) being mature enough to be trusted in production pipelines. This version numbering convention, however, belies the history of a project that has become the standard others follow and has seen extensive adoption in research and industry.

SciPy’s arrival at this point is surprising and somewhat anomalous. When started in 2001, the library had little funding and was written mainly by graduate students—many of them without a computer science education and often without the blessing of their advisors. To even imagine that a small group of ‘rogue’ student programmers could upend the already well-established ecosystem of research software—backed by millions in funding and many hundreds of highly qualified engineers^10–12^—was preposterous.

Yet the philosophical motivations behind a fully open tool stack, combined with an excited, friendly community with a singular focus, have proven auspicious in the long run. They led not only to the library described in this paper, but also to an entire ecosystem of related packages (https://wiki.python.org/moin/NumericAndScientific) and a variety of social activities centered around them (https://wiki.python.org/moin/PythonConferences). The packages in the SciPy ecosystem share high standards of implementation, documentation and testing, and a culture eager to learn and adopt better practices—both for community management and software development.

## Background

Here we capture a selective history of some milestones and important events in the growth of SciPy. Despite what we highlight here, it is important to understand that a project like SciPy is only possible because of the contributions of very many contributors—too many to mention individually, but each bringing an important piece to the puzzle.

Python is an interpreted, high-level, general-purpose computer programming language, designed by Guido van Rossum in the late 1980s, with a dynamic type system and an emphasis on readability and rapid prototyping^13^ (https://github.com/python/cpython). As a general-purpose programming language, it had no special support for scientific data structures or algorithms, unlike many of the other established computation platforms of the time. Yet scientists soon discovered the language’s virtues, such as its ability to wrap C and Fortran libraries, and to then drive those libraries interactively. Scientists could thereby gain access to a wide variety of existing computational libraries without concerning themselves with low-level programming concepts such as memory management.

In 1995, Jim Hugunin, a graduate student at the Massachusetts Institute of Technology, wrote the first message in a new Python Matrix Special Interest Group (Matrix-SIG) mailing list^14^:

“There seems to be a fair amount of interest in the Python community concerning the addition of numeric operations to Python. My own desire is to have as large a library of matrix based functions available as possible (linear algebra, eigenfunctions, signal processing, statistics, etc.). In order to ensure that all of these libraries interoperate, there needs to be agreement on a basic matrix object that can be used to represent arrays of numbers.”

Over the next several months, conversations on that mailing list by, among others, Jim Fulton, Jim Hugunin, Paul Dubois, Konrad Hinsen and Guido van Rossum led to the creation of a package called Numeric with an array object that supported a high number of dimensions. Jim Hugunin explained the utility of Python for numerical computation^15^:

“I’ve used almost all of the available numerical languages at one time or another over the past 8 years. One thing I’ve noticed is that over time, the designers of these languages are steadily adding more of the features that one would expect to find in a general-purpose programming language.”

This remains a distinguishing feature of Python for science and one of the reasons why it has been so successful in the realm of data science: instead of adding general features to a language designed for numerical and scientific computing, here scientific features are added to a general-purpose language. This broadens the scope of problems that can be addressed easily, expands the sources of data that are readily accessible and increases the size of the community that develops code for the platform.

### SciPy begins

By the late 1990s, discussions appeared on Matrix-SIG expressing a desire for a complete scientific data analysis environment^16^. Travis Oliphant, a PhD student at the Mayo Clinic, released a number of packages^17,18^ that built on top of the Numeric array package, and provided algorithms for signal processing, special functions, sparse matrices, quadrature, optimization, fast Fourier transforms and more. One of these packages, Multipack (http://pylab.sourceforge.net/multipack.html), was a set of extension modules that wrapped Fortran and C libraries to solve nonlinear equations and least-squares problems, integrate differential equations and fit splines. Robert Kern, then an undergraduate student (and currently a SciPy core developer), provided compilation instructions under Windows. Around the same time, Pearu Peterson, a PhD student from Estonia, released F2PY^19^, a command line tool for binding Python and Fortran codes, and wrote modules for linear algebra and interpolation. Eric Jones, while a graduate student at Duke University, wrote packages to support his dissertation, including a parallel job scheduler and genetic optimizer. Gary Strangman, a postdoctoral fellow at Harvard Medical School, published several descriptive and inferential statistical routines^20^.

With a rich programming environment and a numerical array object in place, the time was ripe for the development of a full scientific software stack. In 2001, Eric Jones and Travis Vaught founded Enthought Scientific Computing Solutions (now Enthought, Inc.) in Austin, Texas, USA. To simplify the tool stack, they created the SciPy project, centered around the SciPy library, which would subsume all the above-mentioned packages. The new project quickly gained momentum, with a website and code repository^21^ appearing in February, and a mailing list announced^22^ in June 2001. By August 2001, a first release was announced^23^, an excerpt of which is shown in Box 1. In September, the first documentation was published^24^. The first SciPy workshop^25^ was held in September 2002 at Caltech—a single track, two-day event with 50 participants, many of them developers of SciPy and surrounding libraries.

At this point, scientific Python started attracting more serious attention; code that started as side projects by graduate students had grown into essential infrastructure at national laboratories and research institutes. For example, Paul Dubois at Lawrence Livermore National Laboratory (LLNL) took over the maintenance of Numeric and funded the writing of its manual^26^, and the Space Telescope Science Institute (STScI), which was in charge of Hubble Space Telescope science operations, decided to replace their custom scripting language and analysis pipeline with Python^27^. As STScI continued to use Python for an increasingly large portion of the Hubble Space Telescope data analysis pipeline, they encountered problems with the Python numerical array container. Numeric, the original array package, was suitable for small arrays, but not for the large images processed by STScI. With the Numeric maintainer’s blessing, the decision was made to write NumArray^28^, a library that could handle data on a larger scale. Unfortunately, NumArray proved inefficient for small arrays, presenting the community with a rather unfortunate choice. In 2005, Travis Oliphant combined the best elements of Numeric and NumArray, thereby solving the dilemma. NumPy 1.0 was released^29^ in October 2006, paving the way for the reunified scientific Python community to mature.

### SciPy matures

By the middle to late 2000s, SciPy was starting to mature after a long phase of significant growth and adoption. The scope of the SciPy library narrowed, while the breadth of the ecosystem grew through a new type of auxiliary package: the scikit (https://www.scipy.org/scikits.html), a complementary library developed outside SciPy, allowing for more rapid exploration of experimental ideas while maintaining familiar style and development methodology. In SciPy itself, tooling, development, documentation and release processes became more professional. The library was expanded carefully, with the patience affordable in open-source projects and via best practices, which are increasingly common in the scientific Python ecosystem and industry^30^.

Early versions of SciPy had minimal documentation, but this began to change with the 2006 release of a *Guide to NumPy*^1^. In 2007, Sphinx^31^ made it possible to render hypertext and PDF documents automatically from plain text (docstrings) interspersed with Python code, and in 2008, pydocweb^32^ enabled collaborative documentation development in a wiki-like fashion. The SciPy Documentation Project^33,34^ used these tools to complete documentation of SciPy’s user-facing functionality: offering t-shirts to contributors from around the world in exchange for high-quality text, it collected contributions from over 75 people to produce an 884-page manual^35^. Since then, SciPy has remained committed to maintaining high-quality documentation as part of the normal development cycle.

In the early SciPy workshops, recurrent topics reflected the state of development, with emphasis being placed on the underlying array package, plotting, parallel processing, acceleration/wrapping and user interfaces. By 2004, presentations about the application of SciPy to scientific problems began to appear. The event also started to draw in more keynote speakers from outside the community, such as Guido van Rossum (creator of Python, 2006), Ivan Krstić (One Laptop per Child, 2007), Alex Martelli (Google, 2008) and Peter Norvig (Google Research, 2009). The informal workshop grew from a small gathering of core developers into an international conference with hundreds of attendees, increased funding, a published proceedings and scholarships for attending students. By 2010, the US SciPy conference had multiple tracks, and satellite conferences were being organized by volunteers elsewhere, such as EuroSciPy (since 2008) and SciPy India (since 2009). Special sessions and minisymposia dedicated to scientific Python began appearing at many other events. For example, a three-part minisymposium organized for International Conferences on Computational Science and Engineering (CSE) 2009 was featured in *SIAM News*^36^.

In 2007, Python had a strong enough presence in science and engineering that the editors of *IEEE Computing in Science and Engineering* solicited a special issue about Python in science^37^, edited by Paul Dubois. However, Python was still sufficiently niche that the average reader would need additional information to decide whether it would be useful in their own work. The follow-up March/April 2011 Python for Scientists and Engineers special issue^38^ focused more on the core parts of the scientific Python ecosystem^39^ including NumPy^2^, Cython^40^ and Mayavi^41^. Python became so pervasive that journals began publishing domain-specific special issues. For example, in 2015, *Frontiers in Neuroinformatics* published a collection of 25 articles—covering topics including modeling and simulation, data collection, electrophysiology, visualization as well as stimulus generation and presentation—called Python in Neuroscience^42^.

### SciPy today

**Fig. 1:**
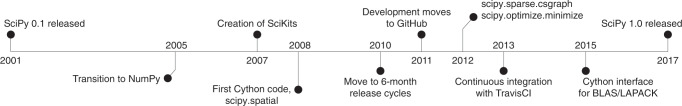
Major milestones from SciPy’s initial release in 2001 to the release of SciPy 1.0 in 2017. Note that SciKits and GitHub have been introduced in the Background section; more information about Cython and SciPy subpackages (for example, scipy.sparse) is available in the ‘Architecture and implementation choices’ section, BLAS/LAPACK support is detailed in the ‘Key technical improvements’ section, and continuous integration is discussed in the ‘Test and benchmark suite’ section.

As of February 2019, the SciPy library consists of nearly 600,000 lines of open-source code organized in 16 subpackages summarized in Box 2. The development team and community currently interact and operate primarily on GitHub, an online version control and task management platform. Over 110,000 GitHub repositories and 6,500 packages depend on SciPy^43^. Some of the major feature highlights from the three years preceding SciPy 1.0 are discussed in the “Key technical improvements” section below, and milestones in its history are highlighted in Fig. 1.

Box 1 Excerpt from the SciPy 0.1 release announcement (typos corrected), posted 20 August 2001 on the Python-list mailing list SciPy is an open-source package that builds on the strengths of Python and Numeric, providing a wide range of fast scientific and numeric functionality. SciPy’s current module set includes the following:

- Special functions (Bessel, Hankel, Airy and others)
- Signal/image processing
- 2D plotting capabilities
- Integration
- ODE solvers
- Optimization (simplex, BFGS, Newton-CG and others)
- Genetic algorithms
- Numeric to C++ expression compiler
- Parallel programming tools
- Splines and interpolation
- Other items

Box 2 Package organization The SciPy library is organized as a collection of subpackages. The 16 subpackages include mathematical building blocks (for example, linear algebra, Fourier transforms, special functions), data structures (for example, sparse matrices, *k*-D trees), algorithms (for example, numerical optimization and integration, clustering, interpolation, graph algorithms, computational geometry) and higher-level data analysis functionality (for example, signal and image processing, statistical methods).

Here we summarize the scope and capabilities of each subpackage. Additional information is available in the SciPy tutorial (https://docs.scipy.org/doc/scipy/reference/tutorial/) and API reference (https://docs.scipy.org/doc/scipy/reference/index.html#api-reference).

cluster

The cluster subpackage contains cluster.vq, which provides vector quantization and *k*-means algorithms, and cluster.hierarchy, which provides functions for hierarchical and agglomerative clustering.

constants

Physical and mathematical constants, including the CODATA recommended values of the fundamental physical constants^119^.

fftpack

Fast Fourier Transform routines. In addition to the FFT itself, the subpackage includes functions for the discrete sine and cosine transforms and for pseudo-differential operators.

integrate

The integrate subpackage provides tools for the numerical computation of single and multiple definite integrals and for the solution of ordinary differential equations, including initial value problems and two-point boundary value problems.

interpolate

The interpolate subpackage contains spline functions and classes, one-dimensional and multi-dimensional (univariate and multivariate) interpolation classes, Lagrange and Taylor polynomial interpolators, and wrappers for FITPACK^53^ and DFITPACK functions.

io

A collection of functions and classes for reading and writing Matlab (https://www.mathworks.com/products/matlab.html), IDL, Matrix Market^120^, Fortran, NetCDF^121^, Harwell-Boeing^122^, WAV and ARFF data files.

linalg

Linear algebra functions, including elementary functions of a matrix, such as the trace, determinant, norm and condition number; basic solver for *Ax* = *b*; specialized solvers for Toeplitz matrices, circulant matrices, triangular matrices and other structured matrices; least-squares solver and pseudo-inverse calculations; eigenvalue and eigenvector calculations (basic and generalized); matrix decompositions, including Cholesky, Schur, Hessenberg, *LU*, *LDL*^T^, *QR*, *QZ*, singular value and polar; and functions to create specialized matrices, such as diagonal, Toeplitz, Hankel, companion, Hilbert and more.

ndimage

This subpackage contains various functions for multi-dimensional image processing, including convolution and assorted linear and nonlinear filters (Gaussian filter, median filter, Sobel filter and others); interpolation; region labeling and processing; and mathematical morphology functions.

misc

A collection of functions that did not fit into the other subpackages. Although this subpackage still exists in SciPy 1.0, an effort is underway to deprecate or relocate the contents of this subpackage and remove it.

odr

Orthogonal distance regression, including Python wrappers for the Fortran library ODRPACK^54^.

optimize

This subpackage includes simplex and interior-point linear programming solvers, implementations of many nonlinear minimization algorithms, a routine for least-squares curve fitting, and a collection of general nonlinear solvers for root-finding.

signal

The signal subpackage focuses on signal processing and basic linear systems theory. Functionality includes convolution and correlation, splines, filtering and filter design, continuous and discrete time linear systems, waveform generation, window functions, wavelet computations, peak finding and spectral analysis.

sparse

This subpackage includes implementations of several representations of sparse matrices. scipy.sparse.linalg provides a collection of linear algebra routines that work with sparse matrices, including linear equation solvers, eigenvalue decomposition, singular value decomposition and LU factorization. scipy.sparse.csgraph provides a collections of graph algorithms for which the graph is represented using a sparse matrix. Algorithms include connected components, shortest path, minimum spanning tree and more.

spatial

This subpackage provides spatial data structures and algorithms, including the *k*-d tree, Delaunay triangulation, convex hulls and Voronoi diagrams. scipy.spatial.distance provides a large collection of distance functions, along with functions for computing the distance between all pairs of vectors in a given collection of points or between all pairs from two collections of points.

special

The name comes from the class of functions traditionally known as special functions, but over time, the subpackage has grown to include functions beyond the classical special functions. A more appropriate characterization of this subpackage is simply useful functions. It includes a large collection of the classical special functions such as Airy, Bessel and others; families of orthogonal polynomials; the Gamma function, and functions related to it; functions for computing the PDF, CDF and quantile function for several probability distributions; information theory functions; combinatorial functions comb and factorial; and more.

stats

The stats subpackage provides a large collection of continuous and discrete probability distributions, each with methods to compute the PDF or PMF, CDF, moments and other statistics, generation of random variates and more; statistical tests, including tests on equality of means/medians/variance (such as the *t*-test) and tests whether a sample is drawn from a certain distribution (such as the Kolmogorov-Smirnov test); measures of correlation, including Pearson’s *r*, Kendall’s *τ*, and Spearman’s *ρ* coefficients; descriptive statistics including trimmed values; kernel density estimation; and transformations of data such as the Box-Cox power transformation.

## Architecture and implementation choices

### Project scope

SciPy provides fundamental algorithms for scientific computing. The breadth of its scope was derived from the guide to available mathematical software (GAMS) classification system^44^. In areas that move relatively slowly, for example, linear algebra, SciPy aims to provide complete coverage. In other areas it aims to provide fundamental building blocks while interacting well with other packages specialized in that area. For example, SciPy provides what one expects to find in a statistics textbook (probability distributions, hypothesis tests, frequency statistics, correlation functions, and more), whereas Statsmodels^45^ provides more advanced statistical estimators and inference methods, scikit-learn^3^ covers machine learning, and PyMC3^46^, emcee^47^ and PyStan (http://mc-stan.org) cover Bayesian statistics and probabilistic modeling. scikit-image^4^ provides image processing capabilities beyond SciPy’s ndimage, SymPy^48^ provides a Python interface for symbolic computation, and sparse.csgraph and spatial offer basic tools for working with graphs and networks compared to specialized libraries such as NetworkX^49^.

We use the following criteria to determine whether to include new functionality in SciPy:

- The algorithm is of relevance to multiple fields of science.
- The algorithm is demonstrably important. For example, it is classic enough to be included in textbooks, or it is based on a peer-reviewed article that has a substantial number of citations.

In terms of software systems and architecture, SciPy’s scope matches NumPy’s: algorithms for in-memory computing on single machines, with support for a wide range of data types and process architectures. Distributed computing and support for graphics processing units (GPUs) were explicitly out of scope at the 1.0 release point, but this has been revised in our roadmap (see Discussion).

### Language choices

According to analysis using the linguist library (https://github.com/github/linguist), SciPy is approximately 50% Python, 25% Fortran, 20% C, 3% Cython and 2% C++, with a dash of TeX, Matlab, shell script and Make. The distribution of secondary programming languages in SciPy is a compromise between a powerful, performance-enhancing language that interacts well with Python (that is, Cython) and the usage of languages (and their libraries) that have proven reliable and performant over many decades.

Fortran, despite its age, is still a high-performance scientific programming language with continued contemporary usage^50^. Thus, we wrap the following excellent, field-tested Fortran libraries to provide Python convenience while benefiting from their performance: QUADPACK^51^ and ODEPACK^52^ for numerical integration and solution of initial value problems; FITPACK^53^, ODRPACK^54^ and MINPACK^55^ for curve-fitting and least-squares minimization; FFTPACK^56,57^ for performing Fourier transforms; ARPACK^58^ for solving eigenvalue problems; ALGORITHM 644 (ref. ^59^) for computing Bessel functions; and CDFLIB^60^ for evaluating cumulative density functions.

Rounding out the top three languages in SciPy is C, which is also extremely well-established over several decades^61^ of scientific computing. The C libraries that we wrap in SciPy include trlib^62^ for optimization, SuperLU^63,64^ for solving sparse linear systems, Qhull^65^ for computational geometry and Cephes (http://www.netlib.org/cephes/) for special functions.

Cython has been described as a creole language that mixes the best parts of Python and lower-level C/C++ paradigms^40^. We often use Cython as a glue between well-established, low-level scientific computing libraries written in C/C++ and the Python interface offered by SciPy. We also use Cython to enable performance enhancements in Python code, especially for cases where heavily used inner loops benefit from a compiled code with static typing.

For implementing new functionality, Python is the still the language of choice. If Python performance is an issue, then we prefer the use of Cython followed by C, C++ or Fortran (in that order). The main motivation for this is maintainability: Cython has the highest abstraction level, and most Python developers will understand it. C is also widely known, and easier for the current core development team to manage than C++ and especially Fortran.

The position that SciPy occupies near the foundation of the scientific Python ecosystem is such that adoption of new languages or major dependencies is generally unlikely; our choices are strongly driven by long-term stability. GPU acceleration, new transpiling libraries and the latest JIT compilation approaches (for example, Numba^66^) are very powerful but have traditionally fallen outside the remit of the main SciPy library. That said, we have recently increased our efforts to support compatibility with some of these options, and our full test suite passed with the PyPy JIT compiler^67^ at the 1.0 release point.

### API and ABI evolution

The API for SciPy consists of approximately 1,500 functions and classes. Our policy for evolving the API over time is that new functionality can be added, while removing or changing existing functionality can only be done if the benefits exceed the (often significant) costs to users and only after giving clear deprecation warnings to those users for at least one year. In general, we encourage changes that improve clarity in the API of the library but strongly discourage breaking backward compatibility, given our position near the base of the scientific Python computing stack.

In addition to the Python API, SciPy has C and Cython interfaces. Therefore, we also have to consider the application binary interface (ABI). This ABI has been stable for a long time, and we aim to evolve it only in a backward-compatible way.

## Key technical improvements

Here we describe key technical improvements made in the last three years.

### Data structures

#### Sparse matrices

**scipy.sparse** offers seven sparse matrix data structures, also known as sparse formats. The most important ones are the row- and column-compressed formats (CSR and CSC, respectively). These offer fast major-axis indexing and fast matrix-vector multiplication, and are used heavily throughout SciPy and dependent packages.

Over the last three years, our sparse matrix handling internals were rewritten and performance was improved. Iterating over and slicing of CSC and CSR matrices is now up to 35% faster, and the speed of coordinate (COO)/diagonal (DIA) to CSR/CSC matrix format conversions has increased. SuperLU^63^ was updated to version 5.2.1, enhancing the low-level implementations leveraged by a subset of our sparse offerings.

From a new features standpoint, scipy.sparse matrices and linear operators now support the Python matrix multiplication (@) operator. We added scipy.sparse.norm and scipy.sparse.random for computing sparse matrix norms and drawing random variates from arbitrary distributions, respectively. Also, we made a concerted effort to bring the scipy.sparse API into line with the equivalent NumPy API where possible.

#### cKDTree

The scipy.spatial.ckdtree module, which implements a space-partitioning data structure that organizes points in *k*-dimensional space, was rewritten in C++ with templated classes. Support was added for periodic boundary conditions, which are often used in simulations of physical processes.

In 2013, the time complexity of the *k*-nearest-neighbor search from cKDTree.query was approximately loglinear^68^, consistent with its formal description^69^. Since then, we enhanced cKDTree.query by reimplementing it in C++, removing memory leaks and allowing release of the global interpreter lock (GIL) so that multiple threads may be used^70^. This generally improved performance on any given problem while preserving the asymptotic complexity.

In 2015, SciPy added the sparse_distance_matrix routine for generating approximate sparse distance matrices between KDTree objects by ignoring all distances that exceed a user-provided value. This routine is not limited to the conventional L2 (Euclidean) norm but supports any Minkowski *p*-norm between 1 and infinity. By default, the returned data structure is a dictionary of keys (DOK)-based sparse matrix, which is very efficient for matrix construction. This hashing approach to sparse matrix assembly can be seven times faster than constructing with CSR format^71^, and the C++ level sparse matrix construction releases the Python GIL for increased performance. Once the matrix is constructed, distance value retrieval has an amortized constant time complexity^72^, and the DOK structure can be efficiently converted to a CSR, CSC or COO matrix to allow for speedy arithmetic operations.

In 2015, the cKDTree dual tree counting algorithm^73^ was enhanced to support weights^74^, which are essential in many scientific applications, for example, computing correlation functions of galaxies^75^.

### Unified bindings to compiled code

#### LowLevelCallable

As of SciPy version 0.19, it is possible for users to wrap low-level functions in a scipy.LowLevelCallable object that reduces the overhead of calling compiled C functions, such as those generated using Numba or Cython, directly from Python. Supported low-level functions include PyCapsule objects, ctypes function pointers and cffi function pointers. Furthermore, it is possible to generate a low-level callback function automatically from a Cython module using scipy.LowLevelCallable.from_cython.

### Cython bindings for BLAS, LAPACK and **special**

SciPy has provided special functions and leveraged basic linear algebra subprograms (BLAS) and linear algebra package (LAPACK)^76^ routines for many years. SciPy now additionally includes Cython^40^ wrappers for many BLAS and LAPACK routines (added in 2015) and the special functions provided in the scipy.special subpackage (added in 2016), which are available in scipy.linalg.cython_blas, scipy.linalg.cython_lapack and scipy.special.cython_special, respectively. When writing algorithms in Cython, it is typically more efficient to call directly into the libraries SciPy wraps rather than indirectly, using SciPy’s Python APIs. These low-level interfaces for Cython can also be used outside of the SciPy codebase to gain access to the functions in the wrapped libraries while avoiding the overhead of Python function calls. This can give performance gains of one or two orders of magnitude for many use cases.

Developers can also use the low-level Cython interfaces without linking against the wrapped libraries^77^. This lets other extensions avoid the complexity of finding and using the correct libraries. Avoiding this complexity is especially important when wrapping libraries written in Fortran. Not only can these low-level wrappers be used without a Fortran compiler, they can also be used without having to handle all the different Fortran compiler ABIs and name mangling schemes.

Most of these low-level Cython wrappers are generated automatically to help with both correctness and ease of maintenance. The wrappers for BLAS and LAPACK are primarily generated using type information that is parsed from the BLAS and LAPACK source files using F2PY^19^, though a small number of routines use hand-written type signatures instead. The input and output types of each routine are saved in a data file that is read at build time and used to generate the corresponding Cython wrapper files. The wrappers in scipy.special.cython_special are also generated from a data file containing type information for the wrapped routines.

Since SciPy can be built with LAPACK 3.4.0 or later, Cython wrappers are only provided for the routines that maintain a consistent interface across all supported LAPACK versions. The standard BLAS interface provided by the various existing BLAS libraries is not currently changing, so changes are not generally needed in the wrappers provided by SciPy. Changes to the Cython wrappers for the functions in scipy.special follow corresponding changes to the interface of that subpackage.

### Numerical optimization

The scipy.optimize subpackage provides functions for the numerical solution of several classes of root finding and optimization problems. Here we highlight recent additions through SciPy 1.0.

#### Linear optimization

A new interior-point optimizer for continuous linear programming problems, linprog with method = ’interior-point’, was released with SciPy 1.0. Implementing the core algorithm of the commercial solver MOSEK^78^, it solves all of the 90+ NETLIB LP benchmark problems^79^ tested. Unlike some interior point methods, this homogeneous self-dual formulation provides certificates of infeasibility or unboundedness as appropriate.

A presolve routine^80^ solves trivial problems and otherwise performs problem simplifications, such as bound tightening and removal of fixed variables, and one of several routines for eliminating redundant equality constraints is automatically chosen to reduce the chance of numerical difficulties caused by singular matrices. Although the main solver implementation is pure Python, end-to-end sparse matrix support and heavy use of SciPy’s compiled linear system solvers—often for the same system with multiple right hand sides owing to the predictor-corrector approach—provide speed sufficient for problems with tens of thousands of variables and constraints.

#### Nonlinear optimization: local minimization

The minimize function provides a unified interface for finding local minima of nonlinear optimization problems. Four new methods for unconstrained optimization were added to minimize in recent versions of SciPy: dogleg, trust-ncg, trust-exact and trust-krylov. All are trust-region methods that build a local model of the objective function based on first and second derivative information, approximate the best point within a local ‘trust region’ and iterate until a local minimum of the original objective function is reached, but each has unique characteristics that make it appropriate for certain types of problems. For instance, trust-exact achieves fast convergence by solving the trust-region subproblem almost exactly, but it requires the second derivative Hessian matrix to be stored and factored every iteration, which may preclude the solution of large problems (≥1,000 variables). In contrast, trust-ncg and trust-krylov are well suited to large-scale optimization problems because they do not need to store and factor the Hessian explicitly, instead using second derivative information in a faster, approximate way. We compare the characteristics of all minimize methods in detail in Table 1, which illustrates the level of completeness that SciPy aims for when covering a numerical method or topic.

**Table 1:** Optimization methods from minimize

	Nelder-Mead	Powell	COBYLA	CG	BFGS	L-BFGS-G	SLSQP	TNC	Newton-CG	dogleg	trust-ncg	trust-exact	trust-Krylov
Version added	0.6*	0.6*	0.6*	0.6*	0.6*	0.6*	0.9	0.6*	0.6*	0.13	0.13	0.19	1.0
Wrapper			✓			✓	✓	✓					✓
First derivatives				✓	✓	✓	✓	✓	✓	✓	✓	✓	✓
Second derivatives					~	~	~	✓	✓	✓	✓	✓	✓
Iterative Hessian factorization								✓	✓		✓		✓
Local convergence				L	S	L	S	S*	S*	Q	S*	Q	S*
Global convergence					✓	✓	✓	✓	✓	✓	✓	✓	✓
Trust region	Neither	LS	TR	LS	LS	LS	LS	LS	LS	TR	TR	TR	TR
Bound constraints						✓	✓	✓	✓				
Equality constraints							✓						
Inequality constraints			✓				✓						
References	^98,99^	^100^	^101–103^	^104,105^	^105^	^106,107^	^108–111^	^112^	^105^	^105,113^	^105,114^	^115,116^	^62,117^

Optimization methods from minimize, which solves problems of the form ${\mathrm{min}}_xf\left(x\right)$, where $x \in {\Bbb R}^n$ and $f:{\Bbb R}^n \to {\Bbb R}$. ‘Version added’ specifies the algorithm’s first appearance in SciPy. Algorithms with version added “0.6*” were added in version 0.6 or before. ‘Wrapper’ indicates whether the implementation available in SciPy wraps a function written in a compiled language (for example, C or FORTRAN). ‘First derivatives’ and ‘second derivatives’ indicate whether first or second order derivatives are required. When ‘second derivatives’ is flagged with ‘~’, the algorithm accepts but does not require second-order derivatives from the user; it computes an approximation internally and uses it to accelerate method convergence.

‘Iterative Hessian factorization’ denotes algorithms that factorize the Hessian in an iterative way, which does not require explicit matrix factorization or storage of the Hessian. ‘Local convergence’ gives a lower bound on the rate of convergence of the iteration sequence once the iterate is sufficiently close to the solution: linear (L), superlinear (S) and quadratic (Q). Convergence rates denoted S* indicate that the algorithm has a superlinear rate for the parameters used in SciPy, but can achieve a quadratic convergence rate with other parameter choices. ‘Global convergence’ is marked for the algorithms with guarantees of convergence to a stationary point (that is, a point x* for which $\nabla f\left( {x^ \ast } \right) = 0$); this is not a guarantee of convergence to a global minimum. ‘Lines-search’ (LS) or ‘trust-region’ (TR) indicates which of the two globalization approaches is used by the algorithm. The table also indicates which algorithms can deal with constraints on the variables. We distinguish among bound constraints ($x^l \le x \le x^u$), equality constraints ($c_{{\mathrm{eq}}}\left( x \right) = 0$) and inequality constraints ($c_{{\mathrm{ineq}}}\left( x \right) \ge 0$).

#### Nonlinear optimization: global minimization

As minimize may return any local minimum, some problems require the use of a global optimization routine. The new scipy.optimize.differential_evolution function^81,82^ is a stochastic global optimizer that works by evolving a population of candidate solutions. In each iteration, trial candidates are generated by combination of candidates from the existing population. If the trial candidates represent an improvement, then the population is updated. Most recently, the SciPy benchmark suite gained a comprehensive set of 196 global optimization problems for tracking the performance of existing solvers over time and for evaluating whether the performance of new solvers merits their inclusion in the package.

### Statistical distributions

The scipy.stats subpackage contains more than 100 probability distributions: 96 continuous and 13 discrete univariate distributions, and 10 multivariate distributions. The implementation relies on a consistent framework that provides methods to sample random variates, to evaluate the cumulative distribution function (CDF) and the probability density function (PDF), and to fit parameters for every distribution. Generally, the methods rely on specific implementations for each distribution, such as a closed-form expression of the CDF or a sampling algorithm, if available. Otherwise, default methods are used based on generic code, for example, numerical integration of the PDF to obtain the CDF. Key recent distributions added to scipy.stats include the histogram-based distribution in scipy.stats.rv_histogram and the multinomial distribution in scipy.stats.multinomial (used, for example, in natural language processing^83^).

### Polynomial interpolators

Historically, SciPy relied heavily on the venerable FITPACK Fortran library by P. Dierckx^53,84^ for univariate interpolation and approximation of data, but the original monolithic design and API for interaction between SciPy and FITPACK was limiting for both users and developers.

Implementing a new, modular design of polynomial interpolators was spread over several releases. The goals of this effort were to have a set of basic objects representing piecewise polynomials, to implement a collection of algorithms for constructing various interpolators, and to provide users with building blocks for constructing additional interpolators.

At the lowest level of the new design are classes that represent univariate piecewise polynomials: PPoly (SciPy 0.13)^85^, BPoly (SciPy 0.13) and BSpline (SciPy 0.19)^86^, which allow efficient vectorized evaluations, differentiation, integration and root-finding. PPoly represents piecewise polynomials in the power basis in terms of breakpoints and coefficients at each interval. BPoly is similar and represents piecewise polynomials in the Bernstein basis (which is suitable, for example, for constructing Bézier curves). BSpline represents spline curves, that is, linear combinations of B-spline basis elements^87^.

In the next layer, these polynomial classes are used to construct several common ways of interpolating data: CubicSpline (SciPy 0.18)^88^ constructs a twice differentiable piecewise cubic function, Akima1DInterpolator and PCHIPInterpolator implement two classic prescriptions for constructing a *C*^1^ continuous monotone shape-preserving interpolator^89,90^.

### Test and benchmark suite

#### Test suite

Test-driven development has been described as a way to manage fear and uncertainty when making code changes^91^. For each component of SciPy, we write multiple small executable tests that verify its intended behavior. The collection of these, known as a ‘test suite’, increases confidence in the correctness and accuracy of the library, and allows us to make code modifications known not to alter desired behavior. According to the practice of continuous integration^92^, all proposed contributions to SciPy are temporarily integrated with the master branch of the library before the test suite is run, and all tests must be passed before the contribution is permanently merged. Continuously monitoring the number of lines of code in SciPy covered by unit tests is one way we maintain some certainty that changes and new features are correctly implemented.

**Fig. 2:**
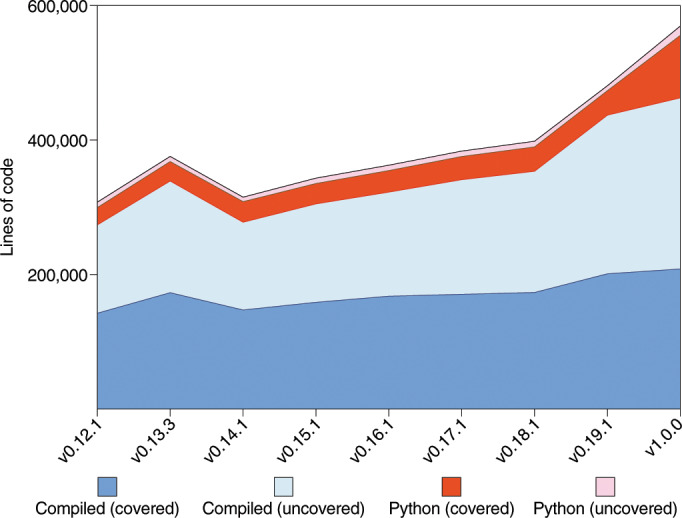
Python and compiled code volume in SciPy over time.

The SciPy test suite is orchestrated by a continuous integration matrix that includes POSIX and Windows (32/64-bit) platforms managed by Travis CI and AppVeyor, respectively. Our tests cover Python versions 2.7, 3.4, 3.5, 3.6, and include code linting with pyflakes and pycodestyle. There are more than 13,000 unit tests in the test suite, which is written for usage with the pytest (https://docs.pytest.org/en/latest) framework. In Fig. 2, we show historical test coverage data generated using a Docker-based approach (https://github.com/tylerjereddy/scipy-cov-track). With the exception of the removal of ∼61,000 lines of compiled code for SciPy v0.14, the volume of both compiled (C, C++ and Fortran) and Python code has increased between releases, as have the number of lines covered by unit tests. Test coverage at the SciPy 1.0 release point was at 87% for Python code according to pytest-cov (https://pypi.org/project/pytest-cov/). Coverage of compiled (C, C++ and Fortran) code was only 45% according to gcov (https://gcc.gnu.org/onlinedocs/gcc/Gcov.html), but the compiled codebase is much more robust than this figure would suggest as the figure does not correct for the inclusion of reputable vendor code, the original library of which is well-tested; generated code, for which full coverage is impractical; and deprecated code, which does not require unit tests. Documentation for the code is automatically built and published by the CircleCI service to facilitate evaluation of documentation changes/integrity.

#### Benchmark suite

**Fig. 3:**
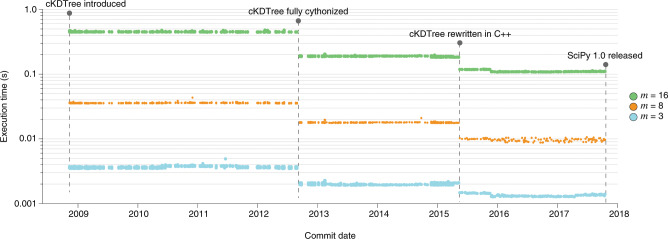
Results of the **scipy.spatial.cKDTree.query** benchmark from the introduction of **cKDTree** to the release of SciPy 1.0. The benchmark generates a *k*-d tree from uniformly distributed points in an *m*-dimensional unit hypercube, then finds the nearest (Euclidean) neighbor in the tree for each of 1,000 query points. Each marker in the figure indicates the execution time of the benchmark for a commit in the master branch of SciPy.

In addition to ensuring that unit tests are passing, it is important to confirm that the performance of the SciPy codebase improves over time. Since February 2015, the performance of SciPy has been monitored with Airspeed Velocity (asv https://github.com/airspeed-velocity/asv). SciPy’s run.py script conveniently wraps asv features such that benchmark results over time can be generated with a single console command. For example, in Fig. 3 we illustrate the improvement of scipy.spatial.cKDTree.query over roughly nine years of project history. The tree used in the benchmark was generated without application of toroidal topology (boxsize = None), and tests were performed by Airspeed Velocity 0.4 using Python 2.7, NumPy 1.8.2 and Cython versions 0.27.3, 0.21.1 and 0.18 (for improved backward compatibility). Substantial performance improvements were realized when cKDTree was fully Cythonized and again when it was rewritten in C++.

## Project organization and community

### Governance

SciPy adopted an official governance document (https://docs.scipy.org/doc/scipy/reference/dev/governance/governance.html) on August 3, 2017. A steering council, currently composed of 18 members, oversees daily development of the project by contributing code and reviewing contributions from the community. Council members have commit rights to the project repository, but they are expected to merge changes only when there are no substantive community objections. The chair of the steering council, Ralf Gommers, is responsible for initiating biannual technical reviews of project direction and summarizing any private council activities to the broader community. The project’s benevolent dictator for life, Pauli Virtanen, has overruling authority on any matter, but is expected to act in good faith and only exercise this authority when the steering council cannot reach agreement.

SciPy’s official code of conduct was approved on October 24, 2017. In summary, there are five specific guidelines: be open to everyone participating in our community; be empathetic and patient in resolving conflicts; be collaborative, as we depend on each other to build the library; be inquisitive, as early identification of issues can prevent serious consequences; and be careful with wording. The code of conduct specifies how breaches can be reported to a code of conduct committee and outlines procedures for the committee’s response. Our diversity statement “welcomes and encourages participation by everyone.”

### Maintainers and contributors

The SciPy project has ~100 unique contributors for every 6-month release cycle. Anyone with the interest and skills can become a contributor; the SciPy contributor guide (https://scipy.github.io/devdocs/dev/contributor/contributor_toc.html) provides guidance on how to do that. In addition, the project currently has 15 active (volunteer) maintainers: people who review the contributions of others and do everything else needed to ensure that the software and the project move forward. Maintainers are critical to the health of the project^93^; their skills and efforts largely determine how fast the project progresses, and they enable input from the much larger group of contributors. Anyone can become a maintainer, too, as they are selected on a rolling basis from contributors with a substantial history of high-quality contributions.

### Funding

The development cost of SciPy is estimated in excess of 10 million dollars by Open Hub (https://www.openhub.net/p/scipy/estimated_cost). Yet the project is largely unfunded, having been developed predominantly by graduate students, faculty and members of industry in their free time. Small amounts of funding have been applied with success: some meetings were sponsored by universities and industry, Google’s Summer of Code program supported infrastructure and algorithm work, and teaching grant funds were used early on to develop documentation. However, funding from national agencies, foundations and industry has not been commensurate with the enormous stack of important software that relies on SciPy. More diverse spending to support planning, development, management and infrastructure would help SciPy remain a healthy underpinning of international scientific and industrial endeavors.

### Downstream projects

The scientific Python ecosystem includes many examples of domain-specific software libraries building on top of SciPy features and then returning to the base SciPy library to suggest and even implement improvements. For example, there are common contributors to the SciPy and Astropy core libraries^94^, and what works well for one of the codebases, infrastructures or communities is often transferred in some form to the other. At the codebase level, the binned_statistic functionality is one such cross-project contribution: it was initially developed in an Astropy-affiliated package and then placed in SciPy afterward. In this perspective, SciPy serves as a catalyst for cross-fertilization throughout the Python scientific computing community.

## Discussion

SciPy has a strong developer community and a massive user base. GitHub traffic metrics report roughly 20,000 unique visitors to the source website between 14 May 2018 and 27 May 2018 (near the time of writing), with 721 unique copies (‘clones’) of the codebase over that time period. The developer community at that time consisted of 610 unique contributors of source code, with more than 19,000 commits accepted into the codebase (GitHub page data).

From the user side, there were 13,096,468 downloads of SciPy from the Python Packaging Index (PyPI)^95^ and 5,776,017 via the default channel of the conda (https://github.com/ContinuumIO/anaconda-package-data) package manager during the year 2017. These numbers establish a lower bound on the total number of downloads by users given that PyPI and conda are only two of several popular methods for installing SciPy. The SciPy website (http://www.scipy.org/), which has been the default citation in the absence of a peer-reviewed paper, has been cited over 3,000 times (https://scholar.google.com/scholar?cites=2086009121748039507). Some of the most prominent uses of or demonstrations of credibility for SciPy include the LIGO-Virgo scientific collaboration that lead to the observation of gravitational waves^96^, the fact that SciPy is shipped directly with macOS and in the Intel distribution for Python^97^, and that SciPy is used by 47% of all machine learning projects on GitHub (https://github.blog/2019-01-24-the-state-of-the-octoverse-machine-learning/).

Nevertheless, SciPy continually strives to improve. The SciPy Roadmap (https://docs.scipy.org/doc/scipy-1.0.0/reference/roadmap.html, https://scipy.github.io/devdocs/roadmap.html), summarized in Table 2, is a continually updated document maintained by the community that describes some of the major directions for improvement for the project, as well as specific limitations and matters that require assistance moving forward. In addition to the items on the roadmap, we are still working to increase the number of SciPy usage tutorials beyond our current 15 section offering. Also, the low-level Cython code in our library (which interacts with C-level code and exposes it for Python usage) could use some measure of modernization, including migration to typed memoryviews to handle NumPy arrays.

**Table 2:** Summary of SciPy Roadmap items following 1.0 release

SciPy subpackage	Summary of change
optimize	A few more high-quality global optimizers
fftpack	Reduce overlap with NumPy equivalent
linalg	Reduce overlap with NumPy equivalent
interpolate	New spline fitting and arithmetic routines
interpolate	New transparent tensor-product splines
interpolate	New non-uniform rational B-splines
interpolate	Mesh refinement and coarsening of B-splines and tensor products
signal	Migrate spline functionality to interpolate
signal	Second order sections update to match capabilities in other routines
linalg	Support a more recent version of LAPACK
ndimage	Clarify usage of the ‘data point’ coordinate model, and add additional wrapping modes
sparse	Incorporate sparse arrays from Sparse package^118^
sparse.linalg	Add PROPACK wrappers for faster SVD
spatial	Add support for (quaternion) rotation matrices
special	Precision improvements for hypergeometric, parabolic cylinder and spheroidal wave functions

A problem faced by many open-source projects is attracting and retaining developers. Although it is normal for some individuals to contribute to a project for a while and then move on, too much turnover can result in the loss of institutional memory, leading to mistakes of the past being repeated, APIs of new code becoming inconsistent with the old code and a drifting project scope. We are fortunate that the SciPy project continues to attract enthusiastic and competent new developers while maintaining the involvement of a small but dedicated old guard. There are contributors who were present in the early years of the project who still contribute to discussions of bug reports and reviews of new code contributions. Our benevolent dictator for life has been with the project for more than 10 years and is still actively contributing code, and the head of our steering council, who also acts as a general manager, is approaching his eleventh anniversary. An additional half dozen or so active developers have been contributing steadily for five or more years. The combination of a committed old guard and a host of new contributors ensures that SciPy will continue to grow while maintaining a high level of quality.

A final important challenge to address is the accommodation of GPU and distributed computing without disrupting our conventional and heavily used algorithm/API infrastructure. Although the exact approach we will adopt across the entire library to leverage these emerging technologies remains unclear, and was not a priority at the 1.0 release point, we now have a concrete implementation of a subpackage that allows for the experimental use of multiple backends, such as GPU-tractable data structures, in the new scipy.fft. This will be described in detail in a future report.

### Reporting Summary

Further information on research design is available in the Nature Research Reporting Summary linked to this article.

## Supplementary information

Reporting Summary

## Data Availability

Raw data for Fig. 2 are available at https://github.com/tylerjereddy/scipy-cov-track, and raw data for Fig. 3 are available at https://github.com/scipy/scipy-articles/tree/master/scipy-1.0/supporting_info/asv_bench/cKDTree.

## Code availability

All SciPy library source code is available in the SciPy GitHub repository, https://github.com/scipy/scipy.

## References

[1] Oliphant, T.E. *Guide to NumPy* 1st edn (Trelgol Publishing USA, 2006).

[2] van der Walt S, Colbert SC, Varoquaux G (2011). The NumPy array: a structure for efficient numerical computation. Comput. Sci. Eng..

[3] Pedregosa F (2011). Scikit-learn: machine learning in Python. J. Mach. Learn. Res..

[4] van der Walt S (2014). scikit-image: image processing in Python. Peer J..

[5] Nitz, A. et al. gwastro/pycbc: PyCBC v1.13.2 release, 10.5281/zenodo.1596771 (27 November 2018).

[6] Vallisneri M, Kanner J, Williams R, Weinstein A, Stephens B (2015). The LIGO Open Science Center. J. Phys. Conf. Ser..

[7] Abbott BP (2016). GW150914: First results from the search for binary black hole coalescence with Advanced LIGO. Phys. Rev. D..

[8] Abbott BP (2017). GW170817: observation of gravitational waves from a binary neutron star inspiral. Phys. Rev. Lett..

[9] The Event Horizon Telescope Collaboration. (2019). First M87 event horizon telescope results. III. Data processing and calibration. Astrophys. J. Lett..

[10] Blanton, K. At Mathworks, support + fun = success: CEO Jack Little believes in power of his workers–and their ideas. *The Boston Globe*, J5 (20 April 1997).

[11] Howell, D. Jack Dangermond’s digital mapping lays it all out. *Investor’s Business Daily* (14 August 2009).

[12] Port, O. Simple solutions. *BusinessWeek*, 24–24 (3 October 2005).

[13] van Rossum, G. *Python/C API Reference Manual*, http://citeseerx.ist.psu.edu/viewdoc/download?doi=10.1.1.211.6702&rep=rep1&type=pdf (2001).

[14] Hugunin, J. The matrix object proposal (very long), https://mail.python.org/pipermail/matrix-sig/1995-August/000002.html (18 August 1995).

[15] Hugunin, J. Extending Python for numerical computation, http://hugunin.net/papers/hugunin95numpy.html (1995).

[16] Oliphant, T. E. Moving forward from the last decade of SciPy. Presentation slides, https://conference.scipy.org/scipy2010/slides/travis_oliphant_keynote.pdf (1 July 2010).

[17] Oliphant, T. E. Some Python modules. *Web Archive*, https://web.archive.org/web/19990125091242/http://oliphant.netpedia.net:80/ (25 January 1999).

[18] Oliphant, T. E. Modules to enhance Numerical Python. *Web Archive*, https://web.archive.org/web/20001206213500/http://oliphant.netpedia.net:80/ (6 December 2000).

[19] Peterson P (2009). F2PY: a tool for connecting Fortran and Python programs. Int. J. Comput. Sci. Eng..

[20] Strangman, G. Python modules. *Web Archive*, https://web.archive.org/web/20001022231108/http://www.nmr.mgh.harvard.edu/Neural_Systems_Group/gary/python.html (2000).

[21] SciPy Developers. SciPy.org. *Web Archive*, https://web.archive.org/web/20010309040805/http://scipy.org:80/ (2001).

[22] Vaught, T. N. SciPy Developer mailing list now online, https://mail.python.org/pipermail/scipy-dev/2001-June/000000.html (2001).

[23] Jones, E. ANN: SciPy 0.10–scientific computing with Python, https://mail.python.org/pipermail/python-list/2001-August/106419.html (2001).

[24] Vaught, T.N. Reference documentation and Tutorial documentation are now available for download as tarballs. *Web Archive* https://web.archive.org/web/20021013204556/http://www.scipy.org:80/scipy/site_content/site_news/docs_released1 (2002).

[25] Vaught, T. N. [ANN] SciPy ‘02 - Python for Scientific Computing Workshop, https://mail.python.org/pipermail/numpy-discussion/2002-June/001511.html (2002).

[26] Ascher, D., Dubois, P. F., Hinsen, K., Hugunin, J. & Oliphant, T. E. An open source project: Numerical Python, 10.5281/zenodo.3599566 (2001).

[27] Greenfield, P. How Python slithered Into astronomy. Presentation, https://conference.scipy.org/scipy2011/slides/greenfield_keynote_astronomy.pdf (2011).

[28] Greenfield, P., Miller, J.T., Hsu, J.T. & White, R.L. numarray: a new scientific array package for Python. *PyCon DC* (2003).

[29] NumPy Developers. v1.0, https://github.com/numpy/numpy/releases/tag/v1.0 (25 October 2006).

[30] Millman, K. J. & Pérez, F. Developing open-source scientific practice. in *Implementing Reproducible Research* (CRC Press) 149–183 (2014).

[31] Brandl, G. & the Sphinx team. Sphinx - Python Documentation Generator, http://www.sphinx-doc.org/en/master/ (2007).

[32] Virtanen, P. et al. pydocweb: a tool for collaboratively documenting Python modules via the web. *Web Archive*, https://code.google.com/archive/p/pydocweb/ (2008).

[33] Harrington, J. The SciPy documentation project. In *Proceedings of the 7th Python in Science Conference* (eds G. Varoquaux, G., Vaught, T. & Millman, K. J.) 33–35 (2008).

[34] van der Walt, S. The SciPy documentation project (technical overview). In *Proceedings of the 7th Python in Science Conference* (eds G. Varoquaux, G., Vaught, T. & Millman, K. J.) 27–28 (2008).

[35] Harrington, J. & Goldsmith, D. Progress report: NumPy and SciPy documentation in 2009. In *Proceedings of the 8th Python in Science Conference* (eds Varoquaux, G., van der Walt, S. & Millman, K. J.) 84–87 (2009).

[36] Pérez, F., Langtangen, H. P. & LeVeque, R. Python for scientific computing. In *SIAM Conference on Computational Science and Engineering,* **42** (5) (2009).

[37] Dubois PF (2007). Python: batteries included. Comput. Sci. Eng..

[38] Millman KJ, Aivazis M (2011). Python for scientists and engineers. Comput. Sci. Eng..

[39] Pérez F, Granger BE, Hunter JD (2011). Python: an ecosystem for scientific computing. Comput. Sci. Eng..

[40] Behnel S (2011). Cython: the best of both worlds. Comput. Sci. Eng..

[41] Ramachandran P, Varoquaux G (2011). Mayavi: 3D visualization of scientific data. Comput. Sci. Eng..

[42] Muller E (2015). Python in neuroscience. Front. Neuroinform..

[43] GitHub. Network dependents - scipy/scipy, https://github.com/scipy/scipy/network/dependents (2019).

[44] Boisvert RF, Howe SE, Kahaner DK (1991). The guide to available mathematical software problem classification system. Commun. Stat. Simul. Comput.

[45] Seabold, S. & Perktold, J. Statsmodels: econometric and statistical modeling with Python. In *Proceedings of the 9th Python in Science Conference* 57–61 (2010).

[46] Salvatier J, Wiecki TV, Fonnesbeck C (2016). Probabilistic programming in Python using PyMC3. PeerJ Comput. Sci..

[47] Foreman-Mackey D, Hogg DW, Lang D, Goodman J (2013). emcee: the MCMC hammer. Publ. Astron. Soc. Pac..

[48] Meurer A (2017). SymPy: symbolic computing in Python. PeerJ Comput. Sci..

[49] Hagberg, A. A., Schult, D. A. & Swart, P. J. Exploring network structure, dynamics, and function using NetworkX. In *Proceedings of the 7th Python in Science Conference*. (eds G. Varoquaux, G., Vaught, T. & Millman, K. J.) 11–15 (2008).

[50] Koelbel, C.H. & Zosel, M.E. *The High Performance FORTRAN Handbook* (MIT Press, 1993).

[51] Piessens, R., de Doncker-Kapenga, E., Uberhuber, C.W. & Kahaner, D.K. *QUADPACK: A Subroutine Package for Automatic Integration* (Springer, 1983).

[52] Hindmarsh, A.C. ODEPACK, a systematized collection of ODE solvers. *Scientific Computing* 55–64 (1983).

[53] Dierckx, P. *Curve and Surface Fitting with Splines* (Oxford Univ. Press, 1993).

[54] Boggs, P.T., Byrd, R.H., Rogers, J.E. & Schnabel, R.B. *User’s Reference Guide for ODRPACK Version 2.01: Software for Weight Orthogonal Distance Regression* (U.S. Department of Commerce, National Institute of Standards and Technology, 1992).

[55] Moré. Jorge J., Garbow, B. S. & Hillstrom, K. E. User guide for MINPACK-1. Report ANL-80–74 (Argonne National Laboratory, 1980).

[56] Swarztrauber, P. N. Vectorizing the FFTs. In *Parallel Computations* (ed. Rodrigue, G.) 51–83 (Academic, 1982).

[57] Swarztrauber PN (1984). FFT algorithms for vector computers. Parallel Comput..

[58] Lehoucq, R.B., Sorensen, D.C. & Yang, C. ARPACK users’ guide: solution of large scale eigenvalue problems with implicitly restarted Arnoldi methods. (Rice University, 1997).

[59] Amos DE (1986). Algorithm 644: A portable package for Bessel functions of a complex argument and nonnegative order. ACM Trans. Math. Softw..

[60] Brown, B., Lovato, J. & Russell, K. CDFLIB, https://people.sc.fsu.edu/~jburkardt/f_src/cdflib/cdflib.html (accessed 6 July 2018).

[61] Kernighan, B. W. & Ritchie, D. M. *The C Programming Language* 2nd edn (Prentice Hall Professional Technical Reference, 1988).

[62] Lenders F, Kirches C, Potschka A (2018). trlib: a vector-free implementation of the GLTR method for iterative solution of the trust region problem. Optim. Methods Softw..

[63] Li, X.S. et al. SuperLU Users’ Guide. Report LBNL-44289 (Lawrence Berkeley National Laboratory, 1999).

[64] Li XS (2005). An overview of SuperLU: algorithms, implementation, and user interface. ACM Trans. Math. Softw..

[65] Barber CB, Dobkin DP, Huhdanpaa H (1996). The Quickhull algorithm for convex hulls. ACM Trans. Math. Softw..

[66] Lam, S. K., Pitrou, A. & Seibert, S. Numba: A LLVM-based Python JIT compiler. In *Proceedings of the Second Workshop on the LLVM Compiler Infrastructure in HPC* 7:1–7:6 (ACM, 2015).

[67] Bolz, C. F., Cuni, A., Fijalkowski, M. & Rigo, A. Tracing the meta-level: PyPy’s tracing JIT compiler. In *Proceedings of the 4th Workshop on the Implementation*, *Compilation, Optimization of Object-Oriented Languages and Programming Systems* 18–25 (ACM, 2009).

[68] VanderPlas, J. Benchmarking nearest neighbor searches in Python, https://jakevdp.github.io/blog/2013/04/29/benchmarking-nearest-neighbor-searches-in-python/ (19 April 2013).

[69] Maneewongvatana, S. & Mount, D. M. Analysis of approximate nearest neighbor searching with clustered point sets. Preprint at https://arxiv.org/pdf/cs/9901013.pdf (1999).

[70] Molden, S. ENH: Enhancements to spatial.cKDTree, https://github.com/scipy/scipy/pull/4374/ (7 January 2015).

[71] Aspnas, M., Signell, A. & Westerholm, J. Efficient assembly of sparse matrices using hashing. In *Applied Parallel Computing. State of the Art in S**cientific Computing* (eds Kagstrom, B. et al.) 900–907 (Springer, 2007).

[72] Cormen, T. H., Stein, C., Rivest, R. L. & Leiserson, C. E. *Introduction to Algorithms* 2nd edn (McGraw-Hill Higher Education, 2001).

[73] Moore A. W. et al. Fast algorithms and efficient statistics: N-point correlation functions. In *Mining the Sky. ESO Astrophysics Symposia (European Southern Observatory)* (eds Banday, A. J., Zaroubi, S. & Bartelmann, M.) 71–82 (Springer, 2001).

[74] Feng, Y. ENH: Faster count_neighour in cKDTree / + weighted input data https://github.com/scipy/scipy/pull/5647 (2015).

[75] Martin AM, Giovanelli R, Haynes MP, Guzzo L (2012). The clustering characteristics of HI-selected galaxies from the 40% ALFALFA survey. Astrophys. J..

[76] Anderson, E. et al. *LAPACK Users’ Guide* 3rd edn (Society for Industrial and Applied Mathematics, 1999).

[77] Henriksen, I. Circumventing the linker: using SciPy’s BLAS and LAPACK within Cython. In *Proceedings of the 14th Python in Science Conference**(SciPy 2015)* (eds Huff, K. & Bergstra, J.) 49–52 (2015).

[78] Andersen, E. D. & Andersen, K. D. (2000) The Mosek interior point optimizer for linear programming: an implementation of the homogeneous algorithm. In *High Performance Optimization* 197–232 (Springer, 2000).

[79] The NETLIB LP test problem set, http://www.numerical.rl.ac.uk/cute/netlib.html (2019).

[80] Andersen ED, Andersen KD (1995). Presolving in linear programming. Math. Program..

[81] Wormington M, Panaccione C, Matney Kevin M, Bowen DK (1999). Characterization of structures from X-ray scattering data using genetic algorithms. Philos. Trans. R. Soc. Lond. A.

[82] Storn R, Price K (1997). Differential evolution — a simple and efficient heuristic for global optimization over continuous spaces. J. Glob. Optim..

[83] Griffiths TL, Steyvers M (2004). Finding scientific topics. Proc. Natl Acad. Sci. USA.

[84] Dierckx, P. *Curve and Surface Fitting with Splines* (Oxford Univ. Press, 1993).

[85] Virtanen, P. ENH: interpolate: rewrite ppform evaluation in Cython, https://github.com/scipy/scipy/pull/2885 (2013).

[86] Burovski, E. add b-splines, https://github.com/scipy/scipy/pull/3174 (27 December 2013).

[87] de Boor, C. *A Practical Guide to Splines* (Springer, 1978).

[88] Mayorov, N. ENH: CubicSpline interpolator, https://github.com/scipy/scipy/pull/5653 (2 January 2016).

[89] Fritsch FN, Carlson RE (1980). Monotone piecewise cubic interpolation. SIAM J. Numer. Anal..

[90] Akima H (1970). A new method of interpolation and smooth curve fitting based on local procedures. J. Assoc. Comput. Mach..

[91] Beck, K. *Test-driven Development: By Example* (Addison-Wesley, 2003).

[92] Silver A (2017). Collaborative software development made easy. Nature.

[93] Eghbal, N. *Roads and Bridges: The Unseen Labor Behind Our Digital Infrastructure* (Ford Foundation, 2016).

[94] The Astropy Collaboration. (2018). The Astropy Project: building an open-science project and status of the v2.0 core package. Astron. J..

[95] Lev, O., Dufresne, J., Kasim, R., Skinn, B. & Wilk, J. pypinfo: view PyPI download statistics with ease, https://github.com/ofek/pypinfo (2018).

[96] Abbott BP (2016). Observation of gravitational waves from a binary black hole merger. Phys. Rev. Lett..

[97] David Liu. The Intel distribution for Python, https://software.intel.com/en-us/articles/intel-optimized-packages-for-the-intel-distribution-for-python (25 August 2017, updated 30 October 2017, accessed 25 July 2018).

[98] Nelder JA, Mead R (1965). A simplex method for function minimization. Comput. J..

[99] Wright, M. H. Direct search methods: once scorned, now respectable. *Pitman Research Notes in Mathematics Series* 191–208 (1996).

[100] Powell MJD (1964). An efficient method for finding the minimum of a function of several variables without calculating derivatives. Comput. J..

[101] Powell, M. J. D. A direct search optimization method that models the objective and constraint functions by linear interpolation. In *Advances in Optimization and Numerical Analysis* (eds Gomez, S. & Hennart, J. P.) 51–67 (Springer, 1994).

[102] Powell MJD (1998). Direct search algorithms for optimization calculations. Acta Numerica.

[103] Powell MJD (2007). A view of algorithms for optimization without derivatives. Math. Today Bull. Inst. Math. Appl..

[104] Polak E, Ribiere G (1969). Note sur la convergence de methodes de directions conjuguees. Rev. française d’informatique et. de. Rech. op.érationnelle.

[105] Nocedal, J. & Wright, S. *Numerical Optimization* 2nd edn (Springer Science & Business Media, 2006).

[106] Byrd RH, Lu P, Nocedal J, Zhu C (1995). A limited memory algorithm for bound constrained optimization. SIAM J. Sci. Comput..

[107] Zhu C, Byrd RH, Lu P, Nocedal J (1997). Algorithm 778: L-BFGS-B: Fortran subroutines for large-scale bound-constrained optimization. ACM Trans. Math. Softw..

[108] Schittkowski K (1983). On the convergence of a sequential quadratic programming method with an augmented Lagrangian line search function. Mathematische Operationsforschung und Statistik. Ser. Optim..

[109] Schittkowski K (1982). The nonlinear programming method of Wilson, Han, and Powell with an augmented Lagrangian type line search function. Part 2: an efficient implementation with linear least squares subproblems. Numer. Math..

[110] Schittkowski K (1982). The nonlinear programming method of Wilson, Han, and Powell with an augmented Lagrangian type line search function. Part 1: convergence analysis. Numer. Math..

[111] Kraft, D. A software package for sequential quadratic programming. Report DFVLR-FR 88–28 (Deutsche Forschungs- und Versuchsanstalt für Luft- und Raumfahrt, 1988).

[112] Nash SG (1984). Newton-type minimization via the Lanczos method. SIAM J. Numer. Anal..

[113] Powell, M. J. D. A new algorithm for unconstrained optimization. *Nonlinear Programming* 31–65 (1970).

[114] Steihaug T (1983). The conjugate gradient method and trust regions in large scale optimization. SIAM J. Numer. Anal..

[115] Conn, A.R., Gould, N.I.M. & Toint, P.L. *Trust Region Methods* (SIAM, 2000).

[116] Moré JJ, Sorensen DC (1983). Computing a trust region step. SIAM J. Sci.Statist. Comput..

[117] Gould NIM, Lucidi S, Roma M, Toint PL (1999). Solving the trust-region subproblem using the Lanczos method. SIAM J. Optim..

[118] Abbasi, H. Sparse: a more modern sparse array library. In *Proceedings of the 17th Python in Science Conference* (eds Akici, F. et al.) 27–30 (2018).

[119] Mohr PJ, Newell DB, Taylor BN (2016). CODATA recommended values of the fundamental physical constants: 2014. J. Phys. Chem. Ref. Data.

[120] Boisvert, R. F., Pozo, R., Remington, K., Barrett, R. F. & Dongarra, J. J. Matrix Market: a web resource for test matrix collections. In *Quality of Numerical Software* 125–137 (Springer, 1997).

[121] Rew R, Davis G (1990). NetCDF: an interface for scientific data access. IEEE Comput. Graph. Appl..

[122] Duff, I.S., Grimes, R.G. & Lewis, J.G. Users’ guide for the Harwell-Boeing sparse matrix collection (release I), http://citeseerx.ist.psu.edu/viewdoc/summary?doi=10.1.1.41.8922 (1992).

