# Author Correction: SciPy 1.0: fundamental algorithms for scientific computing in Python

**DOI:** 10.1038/s41592-020-0772-5

**Published:** 2020-02-24

**Authors:** Pauli Virtanen, Ralf Gommers, Travis E. Oliphant, Matt Haberland, Tyler Reddy, David Cournapeau, Evgeni Burovski, Pearu Peterson, Warren Weckesser, Jonathan Bright, Stéfan J. van der Walt, Matthew Brett, Joshua Wilson, K. Jarrod Millman, Nikolay Mayorov, Andrew R. J. Nelson, Eric Jones, Robert Kern, Eric Larson, C J Carey, İlhan Polat, Yu Feng, Eric W. Moore, Jake VanderPlas, Denis Laxalde, Josef Perktold, Robert Cimrman, Ian Henriksen, E. A. Quintero, Charles R. Harris, Anne M. Archibald, Antônio H. Ribeiro, Fabian Pedregosa, Paul van Mulbregt, SciPy 1.0 Contributors, Aditya Vijaykumar, Alessandro Pietro Bardelli, Alex Rothberg, Andreas Hilboll, Andreas Kloeckner, Anthony Scopatz, Antony Lee, Ariel Rokem, C. Nathan Woods, Chad Fulton, Charles Masson, Christian Häggström, Clark Fitzgerald, David A. Nicholson, David R. Hagen, Dmitrii V. Pasechnik, Emanuele Olivetti, Eric Martin, Eric Wieser, Fabrice Silva, Felix Lenders, Florian Wilhelm, G. Young, Gavin A. Price, Gert-Ludwig Ingold, Gregory E. Allen, Gregory R. Lee, Hervé Audren, Irvin Probst, Jörg P. Dietrich, Jacob Silterra, James T Webber, Janko Slavič, Joel Nothman, Johannes Buchner, Johannes Kulick, Johannes L. Schönberger, José Vinícius de Miranda Cardoso, Joscha Reimer, Joseph Harrington, Juan Luis Cano Rodríguez, Juan Nunez-Iglesias, Justin Kuczynski, Kevin Tritz, Martin Thoma, Matthew Newville, Matthias Kümmerer, Maximilian Bolingbroke, Michael Tartre, Mikhail Pak, Nathaniel J. Smith, Nikolai Nowaczyk, Nikolay Shebanov, Oleksandr Pavlyk, Per A. Brodtkorb, Perry Lee, Robert T. McGibbon, Roman Feldbauer, Sam Lewis, Sam Tygier, Scott Sievert, Sebastiano Vigna, Stefan Peterson, Surhud More, Tadeusz Pudlik, Takuya Oshima, Thomas J. Pingel, Thomas P. Robitaille, Thomas Spura, Thouis R. Jones, Tim Cera, Tim Leslie, Tiziano Zito, Tom Krauss, Utkarsh Upadhyay, Yaroslav O. Halchenko, Yoshiki Vázquez-Baeza

**Affiliations:** 1 University of Jyväskylä, Jyväskylä, Finland; 2 Quansight LLC, Austin, TX USA; 3 Ultrasound Imaging, Mayo Clinic, Rochester, MN USA; 4 Electrical Engineering, Brigham Young University, Provo, UT USA; 5 Enthought, Inc., Austin, TX USA; 6 Anaconda Inc., Austin, TX USA; 7 BioResource and Agricultural Engineering Department, California Polytechnic State University, San Luis Obispo, CA USA; 8 Department of Mathematics, University of California Los Angeles, Los Angeles, CA USA; 9 Los Alamos National Laboratory, Los Alamos, NM USA; 10 Independent researcher, Tokyo, Japan; 11 Higher School of Economics, Moscow, Russia; 12 Independent researcher, Saue, Estonia; 13 Department of Mechanics and Applied Mathematics, Institute of Cybernetics at Tallinn Technical University, Tallinn, Estonia; 14 Berkeley Institute for Data Science, University of California Berkeley, Berkeley, CA USA; 15 Independent researcher, New York, NY USA; 16 School of Psychology, University of Birmingham, Edgbaston, Birmingham UK; 17 Independent researcher, San Francisco, CA USA; 18 Division of Biostatistics, University of California Berkeley, Berkeley, CA USA; 19 WayRay LLC, Skolkovo Innovation Center, Moscow, Russia; 20 Australian Nuclear Science and Technology Organisation, Lucas Heights, NSW Australia; 21 Institute for Learning and Brain Sciences, University of Washington, Seattle, WA USA; 22 College of Information and Computing Sciences, University of Massachusetts Amherst, Amherst, MA USA; 23 Independent researcher, Amsterdam, the Netherlands; 24 Berkeley Center for Cosmological Physics, University of California Berkeley, Berkeley, CA USA; 25 Bruker Biospin Corp., Billerica, MA USA; 26 University of Washington, Seattle, WA USA; 27 Independent researcher, Toulouse, France; 28 Independent researcher, Montreal, Quebec Canada; 29 New Technologies Research Centre, University of West Bohemia, Plzeň, Czech Republic; 30 Department of Mathematics, Brigham Young University, Provo, UT USA; 31 Oden Institute for Computational Engineering and Sciences, The University of Texas at Austin, Austin, TX USA; 32 Independent researcher, Belmont, Massachusetts USA; 33 Space Dynamics Laboratory, North Logan, UT USA; 34 Independent researcher, Logan, Utah USA; 35 Anton Pannekoek Institute, Amsterdam, The Netherlands; 36 Graduate Program in Electrical Engineering, Universidade Federal de Minas Gerais, Belo Horizonte, Brazil; 37 Google LLC, Montreal, Quebec Canada; 38 Google LLC, Cambridge, MA USA; 40 International Centre for Theoretical Sciences, Tata Institute of Fundamental Research, Bengaluru, India; 41 Department of Physics, Birla Institute of Technology and Science, Pilani, India; 42 Independent researcher, Milan, Italy; 43 Institute of Environmental Physics, University of Bremen, Bremen, Germany; 44 Department of Computer Science, University of Illinois at Urbana-Champaign, Urbana, IL USA; 45 Laboratoire Photonique, Numérique et Nanosciences UMR 5298, Université de Bordeaux, Institut d’Optique Graduate School, CNRS, Talence, France; 46 The University of Washington eScience Institute, The University of Washington, Seattle, WA USA; 47 Federal Reserve Board of Governors, Washington, DC, USA; 48 Datadog Inc., New York, NY USA; 49 HQ, Orexplore, Stockholm, Sweden; 50 Statistics Department, University of California - Davis, Davis, CA USA; 51 Emory University, Atlanta, GA USA; 52 Applied BioMath, Concord, MA USA; 53 Department of Computer Science, University of Oxford, Oxford, UK; 54 NeuroInformatics Laboratory, Bruno Kessler Foundation, Trento, Italy; 55 Independent researcher, Chicago, IL USA; 56 Department of Engineering, University of Cambridge, Cambridge, UK; 57 Aix Marseille Univ, CNRS, Centrale Marseille, LMA, Marseille, France; 58 Interdisciplinary Center for Scientific Computing (IWR), Heidelberg University, Heidelberg, Germany; 59 ABB Corporate Research, ABB AG, Ladenburg, Germany; 60 Institut für Mathematische Optimierung, Technische Universität Carolo-Wilhelmina zu Braunschweig, Braunschweig, Germany; 61 Independent researcher, Cologne, Germany; 62 Lawrence Berkeley National Laboratory, Berkeley, CA USA; 63 Institut für Physik, Universität Augsburg, Augsburg, Germany; 64 Applied Research Laboratories, The University of Texas at Austin, Austin, TX USA; 65 Department of Radiology, School of Medicine, University of Cincinnati, Cincinnati, OH USA; 66 Department of Radiology, Cincinnati Children’s Hospital Medical Center, Cincinnati, OH USA; 67 Ascent Robotics Inc., Tokyo, Japan; 68 ENSTA Bretagne, Brest, France; 69 Faculty of Physics, Ludwig-Maximilians-Universität, München, Germany; 70 Excellence Cluster Universe, München, Germany; 71 Independent researcher, Malden, Massachusetts USA; 72 Data Sciences, Chan Zuckerberg Biohub, San Francisco, CA USA; 73 Faculty of Mechanical Engineering, University of Ljubljana, Ljubljana, Slovenia; 74 Sydney Informatics Hub, The University of Sydney, Camperdown, NSW Australia; 75 Instituto de Astrofísica, Pontificia Universidad Católica de Chile, Santiago, Chile; 76 Max Planck Institute for Extraterrestrial Physics, Garching, Germany; 77 University of Stuttgart, Machine Learning and Robotics Lab, Stuttgart, Germany; 78 Department of Electrical Engineering, Universidade Federal de Campina Grande, Campina Grande, Brazil; 79 Department of Computer Science, Kiel University, Kiel, Germany; 80 Planetary Sciences Group and Florida Space Institute and Department of Physics, University of Central Florida, Orlando, FL USA; 81 Independent researcher, Madrid, Spain; 82 Monash Micro Imaging, Monash University, Clayton, VIC Australia; 83 Department of Molecular, Cellular, and Developmental Biology, University of Colorado, Boulder, Boulder, CO USA; 84 Department of Physics and Astronomy, Johns Hopkins University, Baltimore, MD USA; 85 Independent researcher, Munich, Germany; 86 Center for Advanced Radiation Sources, The University of Chicago, Chicago, IL USA; 87 University of Tübingen, Tübingen, Germany; 88 Independent researcher, Rugby, UK; 89 Two Sigma Investments, New York, NY USA; 90 Department of Mechanical Engineering, Technical University of Munich, Garching, Germany; 91 Independent researcher, Berkeley, CA USA; 92 Independent researcher, London, UK; 93 Independent researcher, Berlin, Germany; 94 Intel Corp., Austin, TX USA; 95 Independent Researcher, Horten, Norway; 96 Independent researcher, Daly City, CA USA; 97 D. E. Shaw Research, New York, NY USA; 98 Division of Computational Systems Biology, Department of Microbiology and Ecosystem Science, University of Vienna, Vienna, Austria; 99 Independent researcher, Melbourne, Australia; 100 School of Physics and Astronomy, University of Manchester, Manchester, UK; 101 Electrical and Computer Engineering, University of Wisconsin–Madison, Madison, WI USA; 102 Dipartimento di Informatica, Università degli Studi di Milano, Milan, Italy; 103 Inter-University Centre for Astronomy and Astrophysics, Ganeshkhind, Pune, India; 104 Kavli Institute for the Physics and Mathematics of the Universe, Kashiwa-shi, Japan; 105 Waymo LLC, Mountain View, CA USA; 106 Faculty of Engineering, Niigata University, Nishi-ku, Niigata, Japan; 107 Virginia Polytechnic Institute and State University, Blacksburg, VA USA; 108 Aperio Software, Headingley Enterprise and Arts Centre, Leeds, UK; 109 Independent researcher, Duisburg, Germany; 110 Broad Institute, Cambridge, MA USA; 111 Independent researcher, Gainesville, FL USA; 112 Department of Psychology, Humboldt University of Berlin, Berlin, Germany; 113 Epiq Solutions, Schaumburg, IL USA; 114 Max Planck Institute for Software Systems, Kaiserslautern, Germany; 115 Department of Psychology and Brain Sciences, Dartmouth College, Hanover, NH USA; 116 Jacobs School of Engineering, University of California San Diego, La Jolla, CA USA

**Keywords:** Computational biology and bioinformatics, Biophysical chemistry, Technology

Correction to: *Nature Methods* 10.1038/s41592-019-0686-2, published online 3 February 2020.

In the version of this article initially published online, the corresponding author designation was missing for Matt Haberland and Tyler Reddy. The affiliation for Evgeni Burovski was given as Higher School of Economics; the correct affiliation is National Research University, Higher School of Economics. In Box 1, “SciPy is an open-source package that builds on the strengths of Python and Numeric, providing a wide range of fast scientific and numeric functionality” was used as the box title; this has been moved to the beginning of the box text and a new title has been provided: “Excerpt from the SciPy 0.1 release announcement (typos corrected), posted 20 August 2001 on the Python-list mailing list.” From the original first sentence of this box, “(text following the % symbol indicates that a typo in the original text has been corrected in the version reproduced here)” has been deleted, and “% hanker to Hankel” and “% Netwon to Newton” have been deleted from the ends of the special functions row and the optimization row, respectively. In the first sentence of the ndimage section of Box 2, “nonlinear filter” has been changed to plural. At the end of the first paragraph of the section “SciPy matures,” “The library was expanded carefully, with the patience affordable in open-source projects and via best practices common in industry” has been changed to “The library was expanded carefully, with the patience affordable in open-source projects and via best practices, which are increasingly common in the scientific Python ecosystem and industry.” In Table 2, “Inequality constraint” has been changed to plural. In the “Nonlinear optimization: global minimization” section, “scipy.optimize.differentialevolution” had been changed to “scipy.optimize.differential_evolution.” In the first sentence of the section “Maintainers and contributors,” “SciPy developer guide” has been changed to “SciPy contributor guide” and the URL has been changed from http://scipy.github.io/devdocs/dev/core-dev/index.html to https://scipy.github.io/devdocs/dev/contributor/contributor_toc.html. In Table 2, entries in the first column have been changed from capitalized to lower-case. Finally, a URL in the second paragraph of the Discussion has been changed from https://scholar.google.com/scholar?q=SciPy to https://scholar.google.com/scholar?cites=2086009121748039507. The errors have been corrected in the print, HTML and PDF versions of the article.

**SciPy 1.0 Contributors**

Aditya Vijaykumar, Alessandro Pietro Bardelli, Alex Rothberg, Andreas Hilboll, Andreas Kloeckner, Anthony Scopatz, Antony Lee, Ariel Rokem, C. Nathan Woods, Chad Fulton, Charles Masson, Christian Häggström, Clark Fitzgerald, David A. Nicholson, David R. Hagen, Dmitrii V. Pasechnik, Emanuele Olivetti, Eric Martin, Eric Wieser, Fabrice Silva, Felix Lenders, Florian Wilhelm, G. Young, Gavin A. Price, Gert-Ludwig Ingold, Gregory E. Allen, Gregory R. Lee, Hervé Audren, Irvin Probst, Jörg P. Dietrich, Jacob Silterra, James T Webber, Janko Slavič, Joel Nothman, Johannes Buchner, Johannes Kulick, Johannes L. Schönberger, José Vinícius de Miranda Cardoso, Joscha Reimer, Joseph Harrington, Juan Luis Cano Rodríguez, Juan Nunez-Iglesias, Justin Kuczynski, Kevin Tritz, Martin Thoma, Matthew Newville, Matthias Kümmerer, Maximilian Bolingbroke, Michael Tartre, Mikhail Pak, Nathaniel J. Smith, Nikolai Nowaczyk, Nikolay Shebanov, Oleksandr Pavlyk, Per A. Brodtkorb, Perry Lee, Robert T. McGibbon, Roman Feldbauer, Sam Lewis, Sam Tygier, Scott Sievert, Sebastiano Vigna, Stefan Peterson, Surhud More, Tadeusz Pudlik, Takuya Oshima, Thomas J. Pingel, Thomas P. Robitaille, Thomas Spura, Thouis R. Jones, Tim Cera, Tim Leslie, Tiziano Zito, Tom Krauss, Utkarsh Upadhyay, Yaroslav O. Halchenko and Yoshiki Vázquez-Baeza

